# SARS-CoV-2 Evolution and Patient Immunological History Shape the Breadth and Potency of Antibody-Mediated Immunity

**DOI:** 10.1093/infdis/jiac332

**Published:** 2022-08-03

**Authors:** Maria Manali, Laura A Bissett, Julien A R Amat, Nicola Logan, Sam Scott, Ellen C Hughes, William T Harvey, Richard Orton, Emma C Thomson, Rory N Gunson, Mafalda Viana, Brian Willett, Pablo R Murcia

**Affiliations:** Medical Research Council-University of Glasgow Centre for Virus Research, Glasgow, United Kingdom; Medical Research Council-University of Glasgow Centre for Virus Research, Glasgow, United Kingdom; Medical Research Council-University of Glasgow Centre for Virus Research, Glasgow, United Kingdom; Medical Research Council-University of Glasgow Centre for Virus Research, Glasgow, United Kingdom; Medical Research Council-University of Glasgow Centre for Virus Research, Glasgow, United Kingdom; Medical Research Council-University of Glasgow Centre for Virus Research, Glasgow, United Kingdom; Medical Research Council-University of Glasgow Centre for Virus Research, Glasgow, United Kingdom; Medical Research Council-University of Glasgow Centre for Virus Research, Glasgow, United Kingdom; Medical Research Council-University of Glasgow Centre for Virus Research, Glasgow, United Kingdom; West of Scotland Specialist Virology Centre, NHS Greater Glasgow and Clyde, Glasgow, United Kingdom; Institute of Biodiversity, Animal Health and Comparative Medicine, University of Glasgow, Glasgow, United Kingdom; Medical Research Council-University of Glasgow Centre for Virus Research, Glasgow, United Kingdom; Medical Research Council-University of Glasgow Centre for Virus Research, Glasgow, United Kingdom

**Keywords:** SARS-CoV-2, antibody-mediated immunity, immunological history, virus evolution, virus neutralization

## Abstract

Since the emergence of severe acute respiratory syndrome coronavirus 2 (SARS-CoV-2), humans have been exposed to distinct SARS-CoV-2 antigens, either by infection with different variants, and/or vaccination. Population immunity is thus highly heterogeneous, but the impact of such heterogeneity on the effectiveness and breadth of the antibody-mediated response is unclear. We measured antibody-mediated neutralization responses against SARS-CoV-2_Wuhan_, SARS-CoV-2α, SARS-CoV-2δ, and SARS-CoV-2ο pseudoviruses using sera from patients with distinct immunological histories, including naive, vaccinated, infected with SARS-CoV-2_Wuhan_, SARS-CoV-2α, or SARS-CoV-2δ, and vaccinated/infected individuals. We show that the breadth and potency of the antibody-mediated response is influenced by the number, the variant, and the nature (infection or vaccination) of exposures, and that individuals with mixed immunity acquired by vaccination and natural exposure exhibit the broadest and most potent responses. Our results suggest that the interplay between host immunity and SARS-CoV-2 evolution will shape the antigenicity and subsequent transmission dynamics of SARS-CoV-2, with important implications for future vaccine design.

## Teaser

The breadth and potency of the antibody-mediated response against SARS-CoV-2 is influenced by the exposure type, the number of exposures, and the genotype of the infecting variant. Patients with mixed immunity acquired by vaccination and infection exhibit most effective responses.

Severe acute respiratory syndrome coronavirus 2 (SARS-CoV-2) emerged in December 2019 [1] causing the largest pandemic of the 21st century. Since the start of the pandemic, different viral lineages emerged, exhibiting highly dynamic transmission patterns [2–5]. This is illustrated by the epidemiology of coronavirus disease 2019 (COVID-19) in the United Kingdom since the first introduction of SARS-CoV-2 in late February 2020, when the Wuhan strain (SARS-CoV-2_W_) was first reported. A D614G variant of the Wuhan strain circulated almost exclusively until September of the same year, when the Alpha strain (SARS-CoV-2α) appeared, initially in the southeast of England [2]. By December 2020, SARS-CoV-2α was the dominating variant. At this point in time, the United Kingdom started a COVID-19 vaccination program that took place with unprecedent pace: by 1 July 2021, approximately 47 million people (mainly adults) had received at least 1 vaccine dose [6]. However, during this period, a new variant (Delta, SARS-CoV-2δ) emerged in India, reaching the United Kingdom in April 2021, and quickly became the most prevalent lineage, until November 2021, when the Omicron variant (SARS-CoV-2ο) was introduced and quickly replaced SARS-CoV-2δ. Over a period of approximately 2 years (February 2020 to March 2022) the UK population experienced 4 COVID-19 pandemic waves, each of them caused by a different SARS-CoV-2 variant (Wuhan, Alpha, Delta, and Omicron). In addition, SARS-CoV-2δ and SARS-CoV-2ο infections have been reported in individuals that had been previously vaccinated or infected by preceding variants [7, 8]. As a result, population immunity against SARS-CoV-2 is likely to be highly heterogeneous. The impact of such immunological heterogeneity on SARS-CoV-2 fitness is far from clear. As antibody-mediated immunity is considered a correlate of protection [9, 10], identifying the factors that affect the humoral immune response is key for COVID-19 preparedness and to design more effective vaccines. Our overall objective was to quantify the breadth and potency of antibodies elicited by different immune histories against distinct SARS-CoV-2 variants. To this end, we measured antibody-mediated immunity against SARS-CoV-2_W_, SARS-CoV-2α, SARS-CoV-2δ, and SARS-CoV-2ο using convalescent serum samples from the Glasgow patient population collected between 31 March 2020 and 22 September 2021. Our sampling strategy captured the complex immunological landscape described above and included sera from naive, vaccinated, infected, as well as vaccinated and infected individuals. Importantly, by combining patient metadata (date of positive polymerase chain reaction [PCR]) with virus genomic epidemiology (prevalence of circulating lineages over time) we were able to select confidently serum samples from patients exposed to 3 major variants that circulated in the United Kingdom (Wuhan, Alpha, and Delta).

## METHODS

### Ethics Statement

Ethical approval was provided by National Health Service Greater Glasgow and Clyde (NHSGGC) Biorepository (application 550).

### Serum Samples

Random residual biochemistry serum samples (approximately 41 000) from primary (general practices) and secondary (hospitals) health care settings were collected by the NHSGGC Biorepository between the 31 March 2020 and 22 September 2021. Associated metadata included age, care type, date of sample collection, date of positive PCR result, date of first and second vaccination, and vaccine manufacturer. Seronegative samples were selected based on their enzyme-linked immunosorbent assay (ELISA) results (SARS-CoV-2 spike 1 [S1] or SARS-CoV-2 receptor-binding domain [RBD]) and the absence of a positive PCR test result or record of vaccination. Samples from vaccinated patients were selected based on their ELISA result (SARS-CoV-2 S1 or SARS-CoV-2 RBD), record of vaccination with 1 or 2 doses at least 14 days prior to blood collection, and absence of a positive PCR test result for at least 14 days after sampling. Samples from infected patients had no record of vaccination, were ELISA positive (SARS-CoV-2 S1 or SARS-CoV-2 RBD), and had a positive PCR test result at least 14 days before sample collection. Samples from infected and vaccinated patients were identified by their positive ELISA result, the presence of positive PCR test result and record of vaccination with 1 or 2 doses at least 14 days prior to blood collection. Samples from infected and infected and vaccinated patients were further stratified to infecting variants (based on the date of the positive PCR test), by identifying key time periods during which each variant was most predominant. All serum samples were inactivated at 56°C for 30 minutes before being tested.

### Cells

HEK293T and 293-ACE2 cells were maintained at 37°C, 5% CO_2_, in Dulbecco’s modified Eagle’s medium (DMEM) supplemented with 10% fetal bovine serum, 2 mM L-glutamine, 100 μg/mL streptomycin, and 100 IU/mL penicillin. HEK293 cells were used to produce HEK293-ACE2 target cells by stable transduction with pSCRPSY-hACE2 and were maintained in complete DMEM supplemented with 2 μg/mL puromycin. HEK293T cells were used for the generation of human immunodeficiency virus (HIV) (SARS-CoV-2) pseudotypes.

### IgG Quantification

Immunoglobulin G (IgG) antibodies against SARS-CoV-2 spike and nucleocapsid proteins were measured using an MSD V-PLEX COVID-19 Coronavirus Panel 2 (K15369) kit. Multiplex Meso Scale Discovery electrochemiluminescence (MSD-ECL) assays were performed according to the manufacturer's instructions. Briefly, 96-well plates were blocked at room temperature for at least 30 minutes. Plates were then washed; samples were diluted 1:5000 and added to the plates along with serially diluted reference standard and serology controls. Plates were incubated for 2 hours and further washed. SULFO-TAG detection antibody was added, and plates were incubated for 1 hour. After incubation, plates were washed and read using a MESO Sector S 600 plate reader. Data were generated by Methodological Mind software and analyzed using MSD Discovery Workbench (version 4.0). Results were normalized to standard(s) and expressed as MSD arbitrary units per mL (AU/mL).

### Neutralization Assays

Pseudotype-based neutralization assays were carried out as described previously [11]. HEK293T cells were transfected with the appropriate SARS-CoV-2 spike gene expression vector (Wuhan, Alpha, Delta, or Omicron) together with p8.9171 and pCSFLW72 using polyethylenimine (Polysciences). HIV (SARS-CoV-2) pseudotype-containing supernatants were harvested 48 hours posttransfection, aliquoted, and frozen at –80°C prior to use. Gene constructs bearing the Wuhan (D614G), Alpha (B.1.1.7), Delta (B.1.617.2), and Omicron (B.1.1.529) spike genes were based on the codon-optimized spike sequence of SARS-CoV-2 and generated by GenScript Biotech. Constructs bore the following mutations relative to the Wuhan-Hu-1 sequence (GenBank: MN908947): Wuhan(D614G), D614G; Omicron (BA.1, B.1.1.529), A67V, Δ69–70, T95I, G142D/Δ143–145, Δ211/L212I, ins214EPE, G339D, S371L, S373P, S375F, K417N, N440K, G446S, S477N, T478K, E484A, Q493R, G496S, Q498R, N501Y, Y505H, T547K, D614G, H655Y, N679K, P681H, N764K, D796Y, N856K, Q954H, N969K, L981F; Alpha (B.1.1.7), L18F, Δ69–70, Δ144, N501Y, A570D, P681H, T716I, S982A, D1118H; and Delta (B.1.617.2), T19R, G142D, Δ156–157, R158G, L452R, T478K, D614G, P681R, D950N.

Neutralization efficiency was measured first using a fixed dilution of serum samples in duplicates. Samples with neutralizing activity ≥ 50% relative to the no serum control were then titrated by serial dilutions. Each sample was serially diluted in triplicate from 1:50 to 1:36 450 in complete DMEM, incubated for 1 hour with HIV (SARS-CoV-2) pseudotypes, and plated onto HEK239-ACE2 target cells. After 48 hours, luciferase activity was measured by adding Steadylite Plus chemiluminescence substrate and analyzed using a Perkin Elmer EnSight multimode plate reader. Antibody titers were estimated by interpolating the point at which infectivity had been reduced to 50% of the value for the no serum control samples. Samples that did not have an antibody titer were arbitrary assigned a value of 50 (the lowest dilution available).

### Statistical Analysis

Shapiro-Wilk tests were performed to assess data homoscedasticity. As data were found not to be normally distributed, nonparametric pairwise Wilcoxon rank sum tests were carried out to assess statistically significant differences in antibody levels between groups and viruses. Holm’s method was used to adjust *P* values to account for multiple statistical comparisons. Separate tests were performed for each group when comparing between viruses, and for each virus when comparing between groups. Pairwise comparisons were presented as connected dot plots, highlighting the significance levels of each of the paired comparisons. All analyses and data visualizations were executed using the stats [12] and ggplot2 [13] packages, respectively, from R version 4.0.5.

### Data Availability

Data of each sample, including metadata and results of each assay, are included in the Supplementary Material.

## RESULTS

**Figure 1.**
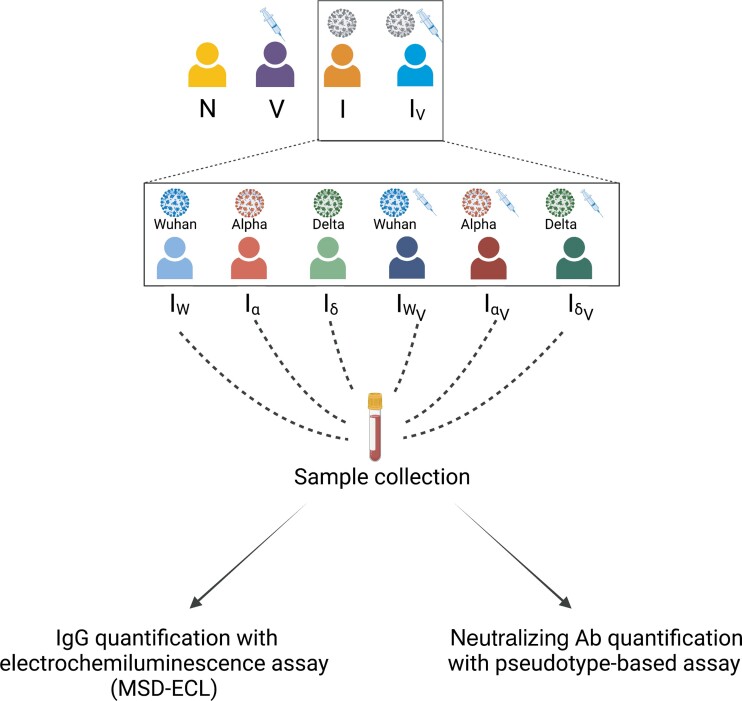
Schematic representation of the study design. Colored silhouettes represent different patient groups (N, naive; V, vaccinated; I, infected; and I_V_, infected and vaccinated). I and I_V_ were further stratified based on the infecting strain (I_W_, infected with SARS-CoV-2_W_; Iα, infected with SARS-CoV-2α; Iδ, infected with SARS-CoV-2δ; I_WV_, vaccinated and infected with SARS-CoV-2_W_; Iα_V_, vaccinated and infected with SARS-CoV-2α; and Iδ_V_, vaccinated and infected with SARS-CoV-2δ). Serum samples were tested for the presence of antibodies against SARS-CoV-2 spike and nucleocapsid, and also tested in virus neutralization assays (see “Methods” section). Abbreviations: AB, antibody; IgG, immunoglobulin G; MSD-ECL, Meso Scale Discovery electrochemiluminescence; SARS-CoV-2, severe acute respiratory syndrome coronavirus 2.

A schematic description of the study is shown in Figure 1. Serum samples (n = 353) from biobanked material that had been collected for serological surveillance studies [11] were selected. The immunological history of each patient at the time of sampling was compiled based on serological status using an ELISA assay that tested for SARS-CoV-2 spike 1 (S1) and receptor binding domain (S1-RBD) [11] together with metadata associated with each clinical specimen. Associated metadata consisted of date of serum collection, SARS-CoV-2 PCR status (including date and result of diagnosis), and vaccination status (including date of vaccination and number of doses). Samples were initially classified in 4 broad groups: naive (N, 30 samples), vaccinated (V, 55 samples) infected (I, 91 samples), and infected and vaccinated (I_V_, 177 samples). Further, sera from the I and I_V_ group were stratified based on the infecting variant as infected-Wuhan (I_W_, 37 samples), infected-Alpha (Iα, 39 samples), infected-Delta (Iδ, 15 samples), infected-Wuhan-vaccinated (I_WV_, 60 samples), infected-Alpha-vaccinated (Iα_V_, 69 samples), and infected-Delta-vaccinated (Iδ_V_, 48 samples). The date of PCR confirmation and the prevalence of each variant at the time of diagnosis was used to infer the most likely infecting variant (see “Methods” section and Supplementary Figure 1). All serum samples were processed as follows: first, they were tested using a multiplex electrochemiluminescence assay against the spike (S), and nucleocapsid (N) proteins to quantify antibody concentrations. Further, serum samples were subjected to virus neutralization assays [11] at a fixed dilution (1:50) using pseudotypes carrying the S glycoprotein of either SARS-CoV-2_W_, SARS-CoV-2α, SARS-CoV-2δ, or SARS-CoV-2ο. Samples that displayed 50% neutralization to at least 1 SARS-CoV-2 variant were titrated as previously described [14].

**Figure 2.**
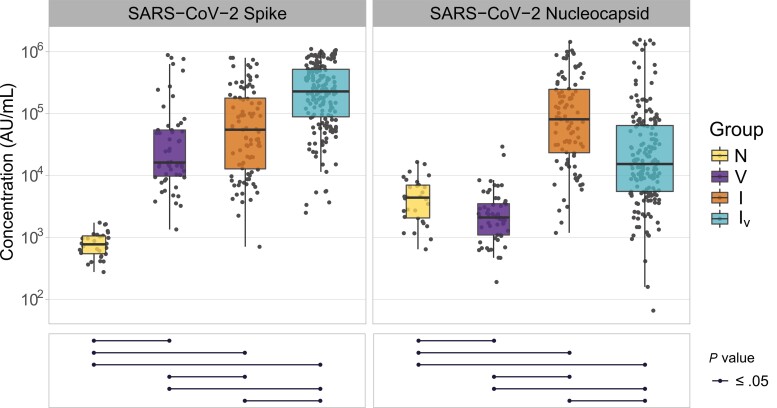
Concentrations of severe acute respiratory syndrome coronavirus 2 (SARS-CoV-2) spike and nucleocapsid antibodies in samples derived from patients with different histories of SARS-CoV-2 exposure. The name of the antigens is shown at the top of each panel. Patient groups are defined as N, naive; V, vaccinated; I, infected; and I_V_, infected and vaccinated. Antibody concentrations are shown in Meso Scale Discovery (MSD) arbitrary units/mL. Box plots display the interquartile range and median values, whiskers display the maximum and minimum values. Significance levels between patient groups were tested using pairwise Wilcoxon test and are shown in bottom panels as connected dot plots.

Quantification of S and N antibody levels for each group of patients is shown in Figure 2. As expected, sera from patients in the naive group (neither vaccinated nor infected) exhibited the lowest levels of anti-S antibodies because they had not been exposed to the spike antigen of SARS-CoV-2. Patients that had been infected displayed higher levels of anti-S antibodies than vaccinated ones, whereas both were significantly lower compared to those observed in sera from patients that had been infected and vaccinated. Sera from patients that had been infected possessed higher levels of anti-N antibodies than those that had been infected and vaccinated (Figure 2), possibly due to a protective effect of vaccination that results in asymptomatic infections and low levels of anti-N antibodies. Of note, vaccinated patients had lower levels of anti-N than naive individuals. Overall, these results are consistent with previous reports suggesting that exposure to SARS-CoV-2 antigens by vaccination and infection results in higher levels of anti-SARS-CoV-2 antibodies than vaccination or infection alone [15–17].

**Figure 3.**
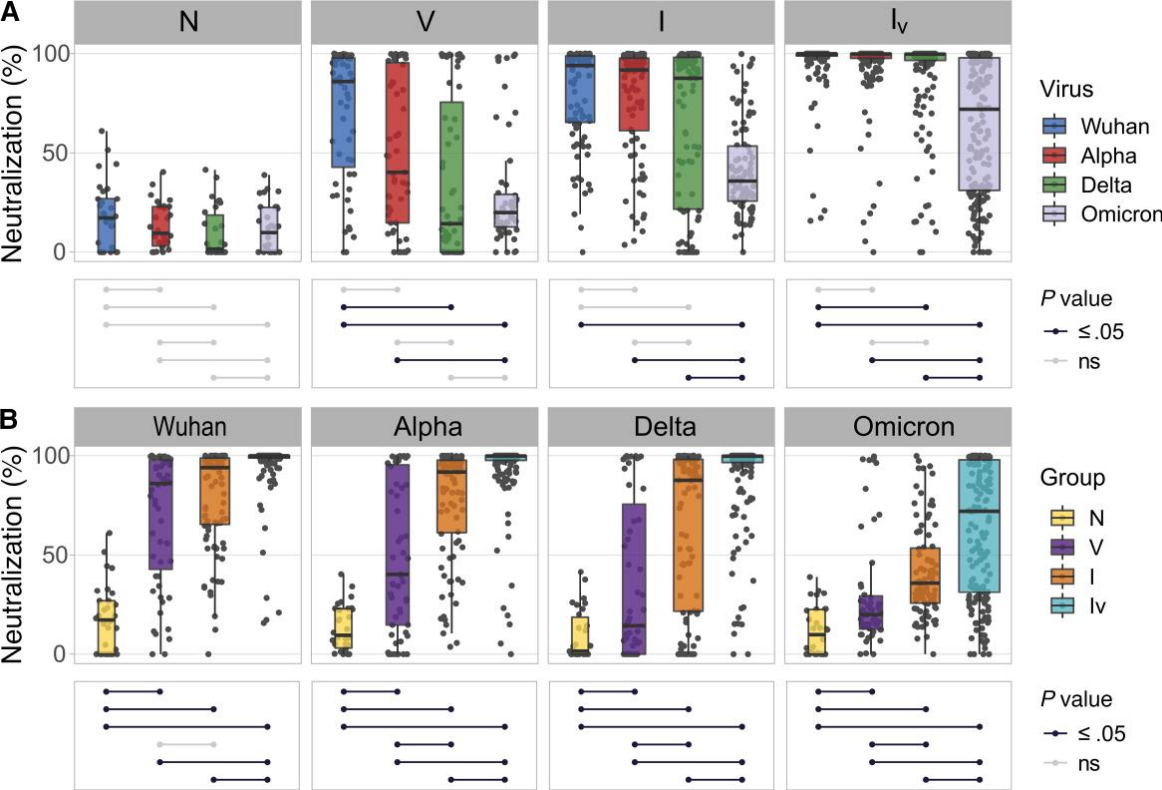
Neutralizing responses elicited against pseudotyped viruses carrying the spike (S) protein of different severe acute respiratory syndrome coronavirus 2 (SARS-CoV-2) variants according to patient exposure history to SARS-CoV-2. Sera from patients were grouped based on immunological histories (N, naive; V, vaccinated; I, infected; and I_V_, infected and vaccinated). Neutralizing activity was measured using Wuhan, Alpha, Delta, and Omicron S glycoprotein bearing HIV(SARS-CoV-2) pseudotypes and plotted per patient group (*A*) and per SARS-CoV-2 S variant (*B*). Neutralization was measured at a fixed dilution (1:50). Each point represents the mean of 2 replicates. Box plots displayed the interquartile range and median values, whiskers display the maximum and minimum values. Significance levels between patient groups or pseudotyped viruses were tested using pairwise Wilcoxon test, and are shown in bottom panels as connected dot plots.

We next measured the neutralization activity of each serum sample at a fixed dilution against SARS-CoV-2_W_, SARS-CoV-2α, SARS-CoV-2δ, and SARS-CoV-2ο using virus pseudotypes. The efficiency of neutralization varied depending on the SARS-CoV-2 variant tested (Figure 3*A*), and the immunological history of the patients (Figure 3*B*). When the chronological order of appearance of each variant is considered, a pattern of neutralization reduction consistent with antigenic drift emerges. This is illustrated by the ladder-like distribution of the median percentage neutralization (Figure 3*A*) and becomes even more evident when neutralization levels are compared between SARS-CoV-2ο and all the other variants, as the former, more evolved S, is neutralized less effectively. A similar trend of neutralization reduction is observed between SARS-CoV-2_W_ and SARS-CoV-2δ in the V and I_V_ groups (Figure 3*A*). We also observed that virus neutralization efficiency increases depending on the number and type of exposures, irrespective of the variant tested (Figure 3*B*). As a result, the I_V_ group exhibited the highest neutralization values against all variants (Figure 3*B*). Supplementary Figure 2 shows differences in virus neutralization due to vaccine type. Within the V group, those that received BNT162b2 (Pfizer/BioNTech) had higher neutralization levels against all SARS-CoV-2 variants than those who received ChAdOx1 (Oxford/AstraZeneca), which was consistent with previously published data [14]. In contrast, no differences in neutralization efficiency against most variants (with the only exception of SARS-CoV-2δ) was observed within the I_V_ group.

**Figure 4.**
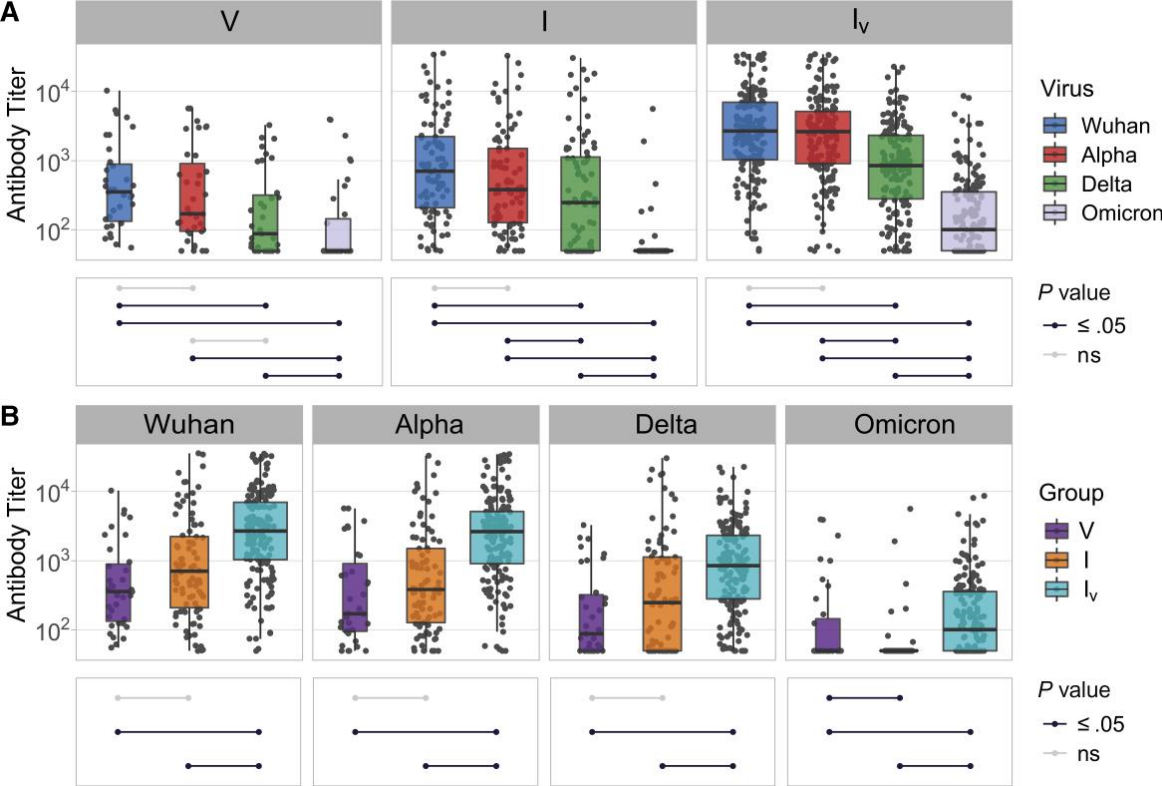
Neutralizing antibody titers against severe acute respiratory syndrome coronavirus 2 (SARS-CoV-2) variants in sera from patients with different histories of SARS-CoV-2 exposure: V, vaccinated; I, infected; and I_V_, infected and vaccinated. Neutralizing activity was measured using Wuhan, Alpha, Delta, and Omicron spike (S) glycoprotein bearing HIV(SARS-CoV-2) pseudotypes and plotted per patient group (*A*) and per SARS-CoV-2 S variant (*B*). Each point represents the mean of 3 replicates. Box plots displayed the interquartile range and median values, whiskers display the maximum and minimum values. Significance levels between patient groups or pseudotyped viruses were tested using pairwise Wilcoxon test and are shown in bottom panels as connected dot plots.

To quantify more accurately the neutralizing potency of the antibody-mediated response among the 4 broad groups (N, V, I, I_V_), we titrated neutralizing antibodies against each variant. Consistent with our previous results, neutralizing titers significantly decreased as SARS-CoV-2 evolved (Figure 4*A*). Indeed, the aforementioned ladder-like effect was even more evident. Also consistent with our previous results, the number and type of antigen exposure events had a significant impact on virus neutralization titers (Figure 4*B*). Patients derived from the I_V_ group displayed significantly higher neutralizing antibody titers compared to every other group across all variants (Figure 4*B*). Supplementary Figure 3 shows the order of infection and vaccination for each patient in the I_V_ group. In turn, infected patients exhibited variable titers against each variant when compared to vaccinated patients: for example, differences between these 2 groups were nonsignificant when SARS-CoV-2_W_, SARS-CoV-2α, and SARS-CoV-2δ were compared. However, patients from the V group displayed significantly higher antibody titers against SARS-CoV-2ο, albeit neutralization efficiency was still very low. Collectively, these results suggest that SARS-CoV-2 antigenic evolution is directional (SARS-CoV-2 evolved to escape antibody-mediated immunity) and that the number and type of exposure events affect the breadth and potency of the antibody-mediated response.

**Figure 5.**
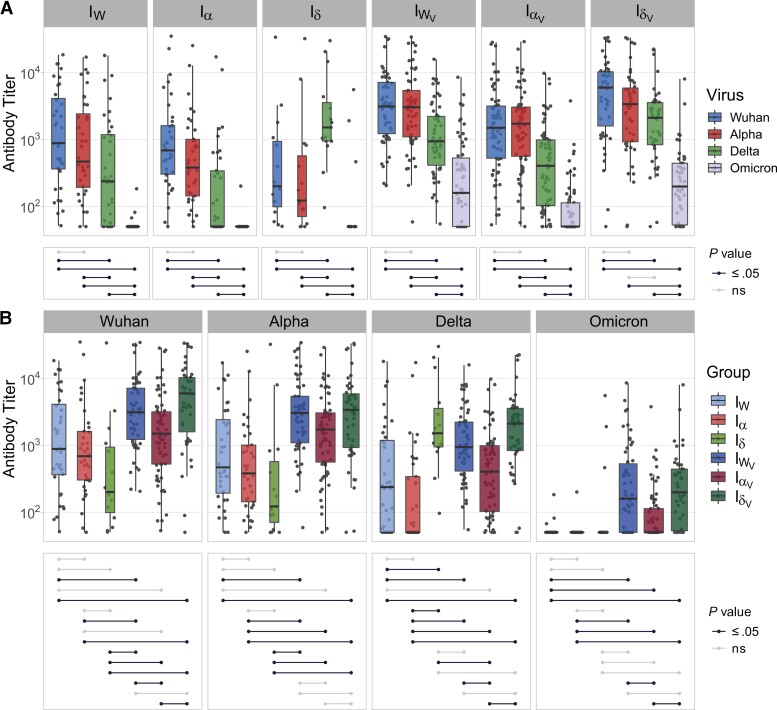
Neutralizing antibody titers against severe acute respiratory syndrome coronavirus 2 (SARS-CoV-2) variants in sera from patients with different histories of SARS-CoV-2 exposure taking into account the infecting SARS-CoV-2 variant: I_W_, infected with SARS-CoV-2_W_; Iα, infected with SARS-CoV-2α; Iδ, infected with SARS-CoV-2δ; I_WV_ infected with SARS-CoV-2_W_ and vaccinated; Iα_V_, infected with SARS-CoV-2α and vaccinated; and Iδ_V_, infected with SARS-CoV-2δ and vaccinated. Neutralizing activity was measured using Wuhan, Alpha, Delta, and Omicron spike glycoprotein bearing HIV(SARS-CoV-2) pseudotypes and plotted per patient group (*A*) and per SARS-CoV-2 S variant (*B*). Each point represents the mean of 3 replicates. Box plots displayed the interquartile range and median values, whiskers display the maximum and minimum values. Significance levels between patient groups or pseudotyped viruses were tested using pairwise Wilcoxon test and are shown in bottom panels as connected dot plots.

**Figure 6.**
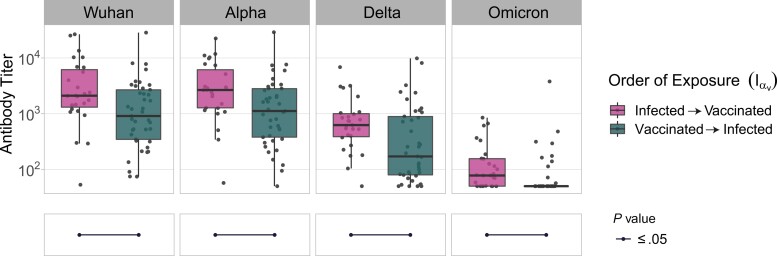
Neutralizing responses against pseudotyped viruses carrying the spike (S) protein of different severe acute respiratory syndrome coronavirus 2 (SARS-CoV-2) variants were titrated in patients that had been either infected and vaccinated or vaccinated and infected with SARS-CoV-2α (Iα_V_). Serum samples were subject to neutralization assays using lentiviruses pseudotyped with the S protein of SARS-CoV-2_W_ (Wuhan), SARS-CoV-2α (Alpha), SARS-CoV-2δ (Delta), or SARS-CoV-2ο (Omicron). Antibody titers were calculated by interpolating the point at which infectivity had been reduced to 50% of the value for the nonserum control samples. Each point represents the mean of 3 replicates, whiskers display the maximum and minimum values. Significance levels between patient groups were tested using pairwise Wilcoxon test and are shown in bottom panels as connected dot plots.

To understand better how humoral immunity is affected by the antigenicity of SARS-CoV-2 variants, we stratified the I and I_V_ groups according to the strains that had infected the patients. This analysis revealed a trend consistent with homologous immunity as sera from patients that had been infected with SARS-CoV-2_W_, SARS-CoV-2α, or SARS-CoV-2δ displayed highest potency against their infecting variants, albeit the differences were not always statistically significant (Supplementary Figure 4*A*). Notably, patients that had been vaccinated and infected with SARS-CoV-2δ showed overall the highest neutralization potency against all variants (Supplementary Figure 4*B*). As this group of patients had been exposed to the most phylogenetically distant antigens (SARS-CoV-2_W_ by vaccination and SARS-CoV-2δ by infection), this result suggests not only that heterologous exposure results in a broader and more effective humoral response but also that the level of antigenic differences between variants affects the potency of the antibody-mediated response. In addition, we observed some differences among patients that had been vaccinated and infected with each SARS-CoV-2 variant: for example, patients that had been infected with SARS-CoV-2α exhibited lower neutralization efficiency against SARS-CoV-2δ than against SARS-CoV-2_W_ or SARS-CoV-2α (Supplementary Figure 4*A*). Titration of neutralizing antibodies enabled us to quantify neutralization biases towards specific SARS-CoV-2 variants. Generally, patients infected by specific variants exhibited significantly different neutralizing titers against other SARS-CoV-2 variants (Figure 5A and 5*B*) and this effect was also evident among vaccinated and infected patients. For example, patients infected with SARS-CoV-2δ exhibited high levels of neutralizing antibodies against SARS-CoV-2δ but significantly lower titers against all other variants (Figure 5*A*), whereas patients infected with SARS-CoV-2_W_ or SARS-CoV-2α displayed similar levels of neutralizing antibodies against SARS-CoV-2_W_ and SARS-CoV-2α but lower levels against SARS-CoV-2δ and even lower against SARS-CoV-2ο (Figure 6). Overall, titers in patients that had been infected only were lower than those measured in patients that had been infected and vaccinated (Figure 5A and 5*B*). The only exception was observed in sera from patients infected with SARS-CoV-2δ, whose neutralizing antibody titers against the homologous antigen was similar in vaccinated and infected patients (Figure 5*B*). Neutralizing antibody responses seemed to display immunological preferences: for example, patients that had been vaccinated and infected with SARS-CoV-2δ exhibited significantly higher neutralization levels against SARS-CoV-2_W_ (the vaccine variant) than SARS-CoV-2δ (the infecting variant), suggesting the stimulation of an anamnestic response. In contrast, patients that had been vaccinated but infected with SARS-CoV-2α showed similar neutralizing antibody titers against SARS-CoV-2_W_ and SARS-CoV-2α. Of note, when neutralizing antibody titers were compared across all patient groups, those vaccinated and infected with SARS-CoV-2δ or infected with SARS-CoV-2_W_ and then vaccinated displayed the highest titers against all variants (Figure 5*B*), consistent with the notion that immunity conferred via infection *and* vaccination results in broader and more potent humoral responses.

As the I_V_ group exhibited the highest antibody titers against all variants, we wanted to determine if the order in which patients were exposed to SARS-CoV-2 (either vaccination first or infection first) played any role in the breadth and potency of antibody mediated neutralization. To test this, we focused on the Iα_V_ group, which exhibited a similar number of patients that had been either infected first (n = 28) or vaccinated first (n = 41). Sera from patients that had been infected first and then vaccinated exhibited significantly higher neutralization efficiency against every variant (Supplementary Figure 5) and also higher titers of neutralizing antibodies (Figure 6). Overall, this result highlights that the type of exposure (vaccination or infection) and the order in which different types of exposure occur have a significant impact on the breadth and potency of humoral immunity against SARS-CoV-2.

## DISCUSSION

Our study shows that the immunological landscape of SARS-CoV-2 is highly heterogeneous and has been shaped by the complex interplay between host immunity and virus evolution. Infection by, or vaccination against, SARS-CoV-2 does not elicit lifelong immunity [18, 19] but instead result in a variety of immune phenotypes, which are likely to influence both transmission dynamics and disease outcomes. We demonstrate that multiple factors influence the breadth and potency of the antibody-mediated response against SARS-CoV-2 and include antigenicity of the exposing pathogen, number of exposures, and exposure type (infection and/or vaccination). While T-cell responses play an important role in SARS-CoV-2 immunity [20], we could not evaluate the impact of cellular-mediated immunity due to the nature of our samples (ie, sera). In line with previous studies, we show that mutations that appeared during SARS-CoV-2 evolution reduce antibody mediated neutralization [21, 22], suggesting that evolution of the spike gene of SARS-CoV-2 is directional and driven by immune selection. This is consistent with reports of reinfections by novel variants [23, 24]. Our results showing that all serum samples exhibited lowest neutralizing activity against pseudoviruses carrying the spike glycoprotein of SARS-CoV-2ο (Figure 4*A* and Figure 5*A*) support this view. As all currently licensed vaccine preparations express the S glycoprotein of SARS-CoV-2_W_, it is expected that the risk of reinfections in vaccinated-only individuals increases as SARS-CoV-2 evolves. Similarly, for infected-only individuals, the risk of reinfection will likely increase as the antigenic distance between the viruses involved in primary and secondary infection increases, and thus is a function of time. Furthermore, the breadth and potency of the antibody response will decrease over time as antibody titers wane (Supplementary Figure 6). Our results also show that more exposure events result in broader and more potent antibody-mediated responses (Figure 3*B* and Figure 4*B*), and this protective effect is also influenced by the antigenic nature of the viruses involved in the primary infection (or vaccination) and subsequent infections. This finding suggests that updates of the vaccine strains (or development of multivalent vaccines) will improve protection against evolving variants, and also that increased transmission of antigenically divergent SARS-CoV-2 viruses among previously exposed individuals will result in future higher levels of protection. We also show that primary infection followed by vaccination results in more potent humoral responses (Figure 6 and Supplementary Figure 5), which indicates that the type and order of exposure events have a significant impact on the breadth and potency of the antibody mediated response. These findings are consistent with recent reports [15, 16, 25]. In a previous study, we showed that increased disease severity in this patient cohort is associated with higher levels of neutralizing antibodies [11]. Because vaccination reduces the severity of disease presentation, it is possible that in vaccinated individuals there is a weaker stimulation of the immune system during infection and as result a lower antibody response. While it is not advisable to promote the acquisition of SARS-CoV-2 immunity by natural infection given the risk of severe disease and/or death due to COVID-19 in naive individuals, our results suggest that vaccines based on live-attenuated viruses might provide increased protection.

In summary, our work underscores the complexity of the immunological landscape of SARS-CoV-2. While our results will inform the development of better epidemiological models to predict the future transmission dynamics of SARS-COV-2 [26], further clinical studies are needed to determine the impact of exposure history on disease presentation to prepare better for the future disease burden of COVID-19 as this disease becomes endemic.

## Supplementary Data

Supplementary materials are available at *The Journal of Infectious Diseases* online. Consisting of data provided by the authors to benefit the reader, the posted materials are not copyedited and are the sole responsibility of the authors, so questions or comments should be addressed to the corresponding author.

## Supplementary Material

jiac332_Supplementary_Data

## Notes

***Acknowledgments*.** We thank Massimo Palmarini for scientific advice and the NHS Greater Glasgow and Clyde Biorepository for providing serum samples.

***Financial support*.** This work was supported by the Medical Research Council of the United Kingdom (grant number MC_UU_12014/9). Funding to pay the Open Access publication charges for this article was provided by a block grant from United Kingdom Research and Innovation (UKRI).
