# Supplementary material for: SARS-CoV-2 evolution and patient immunological history shape the breadth and potency of antibody-mediated immunity
Source: J Infect Dis. 2022 Aug 3:jiac332. doi: 10.1093/infdis/jiac332 (PMC9384671; doi:10.1093/infdis/jiac332)
Supplement: Supplementary file 1 [file jiac332_supplementary_data.zip › Supplementary Data.docx]

**Supplementary Data**

**Supplementary Figure 1**. Number of SARS-CoV-2 variants sequenced in the United Kingdom between March 2020 and September 2021. Variants are colour-coded, and the key is shown on the right of the graph. Vertical dashed lines show the time periods used to select samples as described in the main text and methods.

**Supplementary Figure 2**. Neutralising responses elicited against pseudotyped viruses carrying the S protein of different SARS-CoV-2 variants according to vaccine type. Sera from patients were grouped based on immunological histories (V: vaccinated; I_V_: infected and vaccinated) and considering the vaccine type received (ChAdOx1 [Oxford/AstraZeneca] in red and BNT162b2 [Pfizer/BioNTech] in blue). Serum samples were subject to neutralisation assays using lentiviruses pseudotyped with the S protein of SARS-CoV-2_W_ (Wuhan) SARS-CoV-2α (Alpha), SARS-CoV-2δ (Delta) or SARS-CoV-2o (Omicron). Antibody titres were calculated by interpolating the point at which infectivity had been reduced to 50% of the value for the non-serum control samples. Each point represents the mean of three replicates. Significance levels between patient groups were tested using pairwise Wilcoxon test and are shown in bottom panels as connected dotplots.

**Supplementary Figure 3.** Time and order of infection and vaccination of patients included in the infected and vaccinated (I_V_) group stratified by infecting variant.  Each horizontal line represents a patient. Circles along the lines represent date of sample collection (black circle), date of PCR diagnosis (yellow circles), and date of vaccination (purple circles). Vertical dashed lines show the time periods used to select samples as described in the main text and methods. 

**Supplementary Figure 4**. Neutralising responses elicited against pseudotyped viruses carrying the S protein of different SARS-CoV-2 variants according to patient exposure history. Sera from patients were grouped based on immunological histories taking into account the infecting SARS-CoV-2 variant (I_W_: infected with SARS-CoV-2_W _(light blue), Iα: infected with SARS-CoV-2α (light red), Iδ: infected with SARS-CoV-2δ (light green), I_WV_ infected with SARS-CoV-2_W_ and vaccinated (dark blue), Iα_V_: infected with SARS-CoV-2α (dark red) and vaccinated, Iδ_V_: infected with SARS-CoV-2δ and vaccinated, (dark green)). Neutralising activity was measured using Wuhan (blue), Alpha (red), Delta (green) and Omicron (grey) spike glycoprotein bearing HIV(SARS-CoV-2) pseudotypes and plotted per patient group (A) and per SARS-CoV-2 S variant (B). Neutralisation was measured at a fixed dilution (1:50). Each point represents the mean of two replicates. Boxplots displayed the interquartile range and median values. Significance levels between patient groups or pseudotyped viruses were tested using pairwise Wilcoxon test and are shown in bottom panels as connected dotplots. 

**Supplementary Figure 5.** Neutralising responses elicited against pseudotyped viruses carrying the S protein of different SARS-CoV-2 variants according to the sequential order of exposure (infection and vaccination). Neutralising responses against pseudotyped viruses carrying the S protein of different SARS-CoV-2 variants were measured at a fixed dilution (1:50) in patients that had been either infected and vaccinated (pink) or vaccinated and infected (seagreen), with SARS-CoV-2α. Serum samples were subject to neutralisation assays using lentiviruses pseudotyped with the S protein of SARS-CoV-2_W_ (Wuhan) SARS-CoV-2α (Alpha), SARS-CoV-2δ (Delta) or SARS-CoV-2o (Omicron). Each point represents the mean of three technical replicates. Significance levels between patient groups were tested using pairwise Wilcoxon test and are shown in bottom panels as connected dotplots.

**Supplementary Figure 6.** Patterns of antibody levels over time. (A) Serum samples were subject to neutralisation assays using lentiviruses pseudotyped with the S protein of SARS-CoV-2_W_ (Wuhan) SARS-CoV-2α (Alpha), SARS-CoV-2δ (Delta) or SARS-CoV-2o (Omicron). Antibody titres were calculated by interpolating the point at which infectivity had been reduced to 50% of the value for the non-serum control samples and plotted based on the time between exposure (vaccination or confirmed PCR diagnosis) and serum sample collection. (B) Concentrations of SARS-CoV-2 Spike and Nucleocapsid antibodies in serum samples derived from patients with types of SARS-CoV-2 exposure. Antibody concentrations are shown in MSD arbitrary units/ml and plotted based on time between exposure (vaccination or confirmed PCR diagnosis) and serum sample collection. Exposure type is defined as infection (yellow dots) and vaccination (purple dots). 

**Supplementary Data File.** Assay results and metadata of each serum sample used in this study.
